# Diagnostics in Late Periprosthetic Infections—Challenges and Solutions

**DOI:** 10.3390/antibiotics13040351

**Published:** 2024-04-11

**Authors:** Florian Hubert Sax, Marius Hoyka, Benedikt Paul Blersch, Bernd Fink

**Affiliations:** 1 Department of Joint Replacement, General and Rheumatic Orthopaedics, Orthopaedic Clinic Markgröningen gGmbH, Kurt-Lindemann-Weg 10, 71706 Markgröningen, Germany; florian.sax@rkh-gesundheit.de (F.H.S.); marius.hoyka@rkh-gesundheit.de (M.H.); benedikt.blersch@rkh-gesundheit.de (B.P.B.); 2 Orthopaedic Department, University Hospital Hamburg-Eppendorf, Martinistrasse 52, 20246 Hamburg, Germany

**Keywords:** periprosthetic joint infection, diagnosis, review

## Abstract

The rising number of arthroplasties is combined with a rising number of periprosthetic joint infections, which leads to life-concerning consequences for the patients, including extended antibiotic treatment, further surgery and increased mortality. The heterogeneity of the symptoms and inflammatory response of the patients due to, e.g., age and comorbidities and the absence of a single diagnostic test with 100% accuracy make it very challenging to choose the right parameters to confirm or deny a periprosthetic joint infection and to establish a standardized definition. In recent years, additional diagnostic possibilities have emerged primarily through the increasing availability of new diagnostic methods, such as genetic techniques. The aim of the review is to provide an overview of the current state of knowledge about the various tests, including the latest developments. The combination of different tests increases the accuracy of the diagnosis. Each physician or clinical department must select the tests from the available methods that can be best implemented for them in organizational and technical terms. Serological parameters and the cultivation of the samples from aspiration or biopsy should be combined with additional synovial tests to create an accurate figure for the failure of the prosthesis, while imaging procedures are used to obtain additional information for the planned therapeutic procedure.

## 1. Introduction

Arthroplasty is a standard procedure that has become an integral part of everyday clinical practice. By 2040, the number of operations for hip and knee joint replacements is expected to increase by up to 45% [1]. At the same time, the number of implant-associated complications will increase. In particular, periprosthetic infection is a dreaded complication with potentially life-threatening consequences and a 3.2–3.7-fold increase in mortality rate compared to patients who do not develop an infection following joint replacement [2].

As a result of the increasing number of arthroplasty procedures, revision surgery and the associated diagnostic procedures are becoming more and more important. The correct diagnosis of aseptic or septic revisions is crucial, because the treatment concept for each is fundamentally different.

Effective treatment of a periprosthetic infection therefore begins with an accurate diagnosis. The first attempts to create a classification system for periprosthetic infections began in 1975, when Coventry introduced the distinction between early and late infections [3].

A similar classification system based on the time interval between implantation and infection manifestation was established by Tsukayama in 1996, in which both contamination and hematogenous infection were integrated into the scheme [4].

There have been several attempts to revise this classification, but so far, no time interval has been established that is valid for a uniform differentiation between early and late infections. In particular, the degree of maturity of the biofilm has a therapeutic consequence, whereby the time until the formation of an intact, mature biofilm is approximately 3–4 weeks [5]. Therefore, it is not the distinction between early and late infection that is of decisive importance, but rather the distinction between acute and chronic infection. The interval between the onset of symptoms and the start of treatment plays a decisive role here. The reason for this distinction is that periprosthetic joint infections (PJI) with an immature biofilm (within 4 weeks) can often be successfully treated with debridement and retention of the prosthesis (so-called DAIR) [6,7].

While the diagnosis of early infection (up to 4 weeks after implantation) is clearly characterized by clinical symptoms and indicators of inflammation in the serum and joint aspirate, the diagnosis of late periprosthetic infection represents a considerable challenge. This is also reflected in the number of scientific publications concerning the diagnosis of late periprosthetic infection. From 1998 to 2018 alone, 3200 publications were published on this topic, and these were cited 12,000 times in 2018 alone [8].

The bottom line of all these publications is that there is no single diagnostic test with 100% accuracy that can confirm or rule out a late periprosthetic infection, but that, in contrast, there are many different tests that can be used to form an overall picture of whether a periprosthetic infection is present or not [9].

The reason for this is the heterogeneity of the underlying disease because, although periprosthetic infection always has an inflammatory component, patients differ in their inflammatory response, making it very challenging to use individual biomarkers to establish a standardized definition [10].

The Academy of Orthopaedic Surgeons (AAOS) developed an algorithm for diagnosing perprosthetic infections in 2010, utilizing C-reactive protein (CRP) with a threshold of 10 mg/L as a screening parameter. This approach can result in the PJI being overlooked in approximately 15% of the infected cases [10]. Consequently, a modification of this algorithm was implemented in 2013, and to this day, CRP remains as a part of current diagnostic algorithms for diagnosing periprosthetic infections [11].

**Figure 1:**
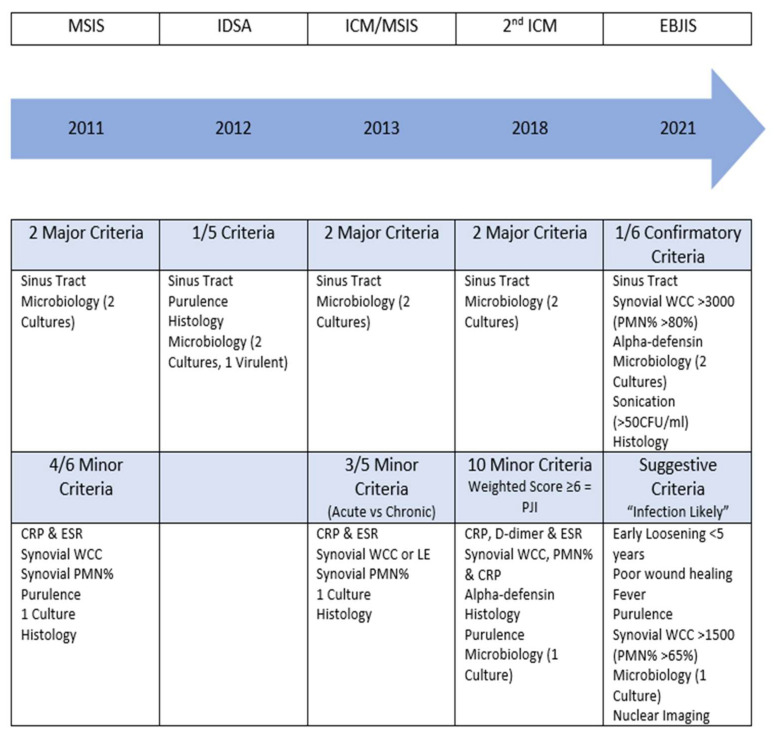
The evolution of the definitions of periprosthetic joint infection classification with criteria. Reproduced from McNally et al. [12].

**Figure 2:**
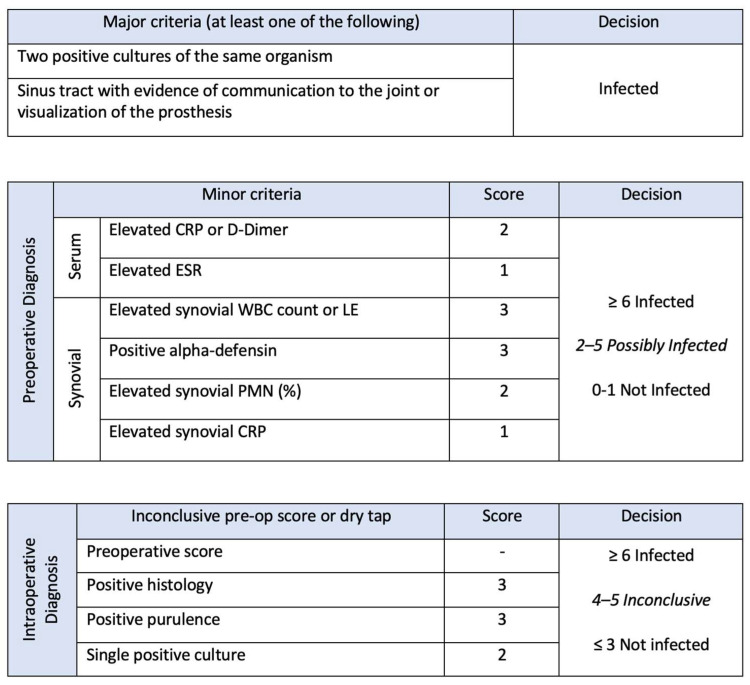
The criteria from the 2018 International Consensus Meeting for the diagnosis of PJI, there are two major criteria and the differentiation between intraoperative and preoperative diagnostic tools. CRP, C-reactive protein; ESR, erythrocyte sedimentation rate; LE, leukocyte esterase; PMN, polymorphonuclear; WBC, white blood cell. Reproduced from Parvizi et al. [15].

The evolution of the definition of periprosthetic infection (Figure 1) took several years, the first definition was established by the Musculoskeletal Infection Society (MSIS) in 2011. Two main criteria and six secondary criteria were specified, whereby a periprosthetic infection was assumed if at least one of the main criteria or four of the six secondary criteria were met [12]. One year later, the Infectious Diseases Society of America (IDSA) published a simplified definition based on the presence of at least one of five criteria [13]. These definitions were revised in 2013 by the first International Conference on Musculoskeletal Infection (ICM) [14] and redefined again in 2018 (Figure 2) [15]. In addition to increasing specificity and sensitivity, the aim of these revisions was to integrate the minor criteria with the weighting of the individual parameters. In addition to improved decision-making for planning the therapeutic procedure, a basic structure was created for the continuation of specific scientific studies.

The following two issues in particular proved to be fundamentally difficult. Firstly, these definitions repeatedly highlighted the weakness in the detection of “low-grade” infections, as these are associated with a significantly less pronounced inflammatory reaction. And secondly, all definitions had in common that they sought to arrive at a binary clinical outcome (infected or not infected) using tests that have neither 100% specificity nor sensitivity.

**Figure 3:**
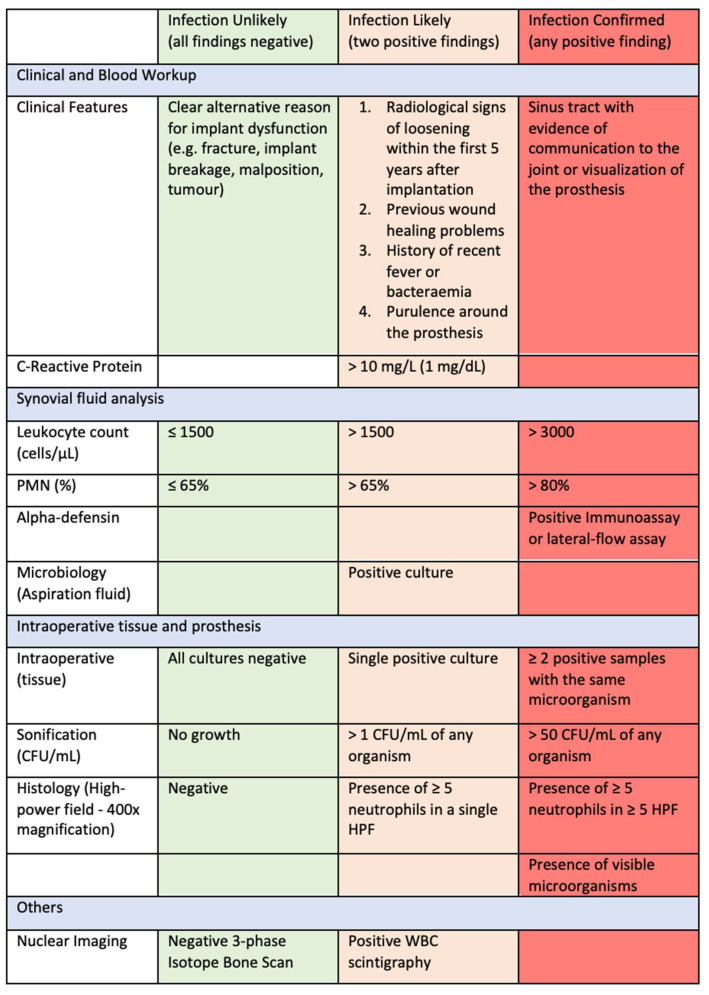
The three groups from the EBJIS diagnosis of the periprosthetic joint infection. Reproduced from McNally et al. [9].

In response to this problem, a new definition was published by the European Bone Joint Infection Society (EBJIS) in 2021 (Figure 3), in which three different groups were defined (infection unlikely, infection likely and infection confirmed) [9]. This definition is supported by the MSIS, the European Society of Clinical Microbiology and Infectious Diseases (ESCMID) and the Study Group for Implant-Associated Infections (ESGIAI). Particular consideration was given to the fact that there are a number of tests that have a very high sensitivity with low specificity (C-reactive protein, scintigraphy) and make an infection probable but cannot prove an infection overall. On the other hand, highly specific markers (fistula formation, detection of two positive samples with the same pathogen) are not present in the majority of reported periprosthetic infections.

Subsequent to diagnosis, the management of periprosthetic joint infection poses the next challenge, with current treatment algorithms determining the type of surgery, single or staged approach, and perioperative antibiotic therapy [16].

## 2. Diagnostic Categories

### 2.1. Clinical Examination

The clinical presentation of periprosthetic infection plays a subordinate role in previous publications for diagnostic guidelines. In everyday clinical practice, however, it is of great importance as it is cost-neutral, non-invasive and easy to handle, and therefore provides the basic building blocks for extending the diagnostic process. Often, there are rather non-specific symptoms such as pain, limited mobility or functional deficits, which can also result from incorrect implantation, abrasion-related aseptic loosening or muscular insufficiency, for example, while fistula formation, local erythema and swelling have a very high specificity but a low sensitivity [17].

However, pain in particular is the clinically guiding symptom in more than 90% of cases and should always lead to further investigation [18].

### 2.2. Imaging Procedures

The first imaging study that is almost always used when a periprosthetic infection is suspected is the X-ray. It is mainly used to rule out mechanical complications such as periprosthetic fractures and dislocations, and it has low sensitivity and specificity. The observable changes are often not yet visible in the early phase of an infection and only manifest themselves during chronic processes. The visualization of a rapid migration of the prosthesis (at least 2 mm within 6–12 months), rapidly progressive or multifocal periprosthetic osteolysis or periosteal reactions and periarticular ossifications may indicate an infection. In addition, generalized bone resorption, excessive sclerosis, fracture of the bone cement and transcortical sinus tracts may occur [19,20]. It is particularly useful to compare the radiographs with previous images [21]. Unfortunately, there is a high degree of overlap in these signs between periprosthetic infections, aseptic loosening and loosening induced by abraded particles. Neither CT, MRI nor ultrasound are included in the current diagnostic catalogs of ICM, MSIS and EBJIS [9,12,15].

The above-mentioned changes to the bone can be further specified by CT, and the additional information on the bony status can be included in the planning of a surgical revision. In addition, fluid accumulations and sinus tracts can also be visualized by soft tissue imaging [22].

As well as improving the visualization of the soft tissue situation, MRI can also reveal changes in bone metabolism as markers for the onset of infection. In addition, well-communicating abscess formations (e.g., psoas abscesses) can be visualized. The artefacts caused by the prosthesis represent a particular problem with MRI. These can be reduced by new methods such as metal artifact reduction sequences (MARS), slice encoding for metal artifact correction (SEMAC), and multiacquisition with variable-resonance image combination (MAVRIC). This increases the benefit of a completed MRI [23,24].

In summary, it can be said that CT and MRI play a subordinate role in the diagnosis of periprosthetic infection and are mainly used to obtain additional information for the planned therapeutic procedure [20,25].

### 2.3. Nuclear Imaging Techniques

The importance of nuclear imaging in the diagnosis of periprosthetic infections is increasing, with the result that it was included in the diagnostic criteria for the first time by the EBJIS 2021 [9]. It is based on the accumulation of various agents (radiolabeled cells, peptides, antibodies or 18-fluorodeoxyglucose [FDG]) along the infected prosthesis. They primarily play a role as an exclusion criterion, as they have very high specificity. For example, a negative three-phase scintigraphy (2 years after THA or 5 years after TKA) can virtually rule out an infection [26]. In addition, newer methods, such as the enrichment of isotopes in leukocyte scintigraphy over a 20 h period, can make the diagnosis of an infection probable [27]. The existing references show that nuclear imaging is particularly beneficial in patients with a high pre-test probability of infection, while it has no clear additional value in patients with a low a priori probability [28]. Other methods, such as single photon emission CT (SPECT) and fluorodeoxyglucose positron emission tomography (FDG-PET), have not been shown to have superior outcomes either [29]. The fundamental problem is that physiological remodeling processes after prosthesis implantation as well as aseptic and abrasion-induced loosening are associated with increased periprosthetic metabolism, and the methodology is currently not widely available and extremely cost-intensive.

In the future, new methods such as gallium-68 (68Ga)-labeled fibroblast activation protein inhibitor positron emission tomography/computer tomography (68Ga-FAPI PET/CT) may offer a promising approach [30]. Fibroblast activation plays an important role in inflammation, infection and immune response, which is also found in chronic infections [31].

In this context, the visualization of periprosthetic infections differs significantly from that of aseptic loosening [32]. Pathogen-specific hybrid tracers (e.g., 99mTc-UBI29-41-Cy5 for Staph. aureus infections) also represent a new diagnostic option and can also detect metabolically less active bacteria in the biofilm [33].

### 2.4. Serum Biomarkers

**Figure 4:**
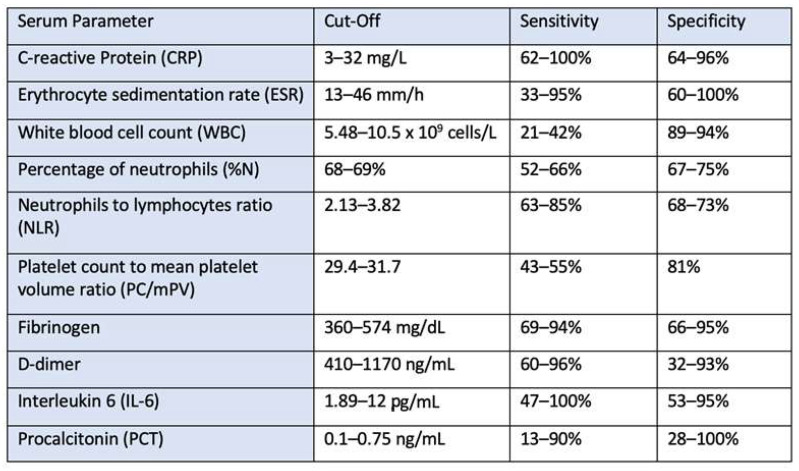
Sensitivity and specificity of the different serum parameters with cut-off levels. Reproduced from Sigmund et al. [35].

Serum biomarkers such as CRP, erythrocyte sedimentation rate, leukocyte count, neutrophil granulocyte count, neutrophil granulocyte to lymphocyte ratio, fibrinogen, D-dimers, interleukin-6 and procalcitonin are inexpensive, diagnostic tools that are almost always available and give rapid results for the diagnosis of periprosthetic infections. These are systemic biomarkers and therefore not specific for the diagnosis of periprosthetic infection. Serum C-reactive protein (CRP) and fibrinogen are the most accurate markers [34,35]. CRP is an acute phase protein and is used as a general parameter for inflammatory reactions. Formed in the liver, it is independent of the cause of inflammation and is elevated in infections, but also in autoimmune diseases, cancer and renal failure [36]. There is no precise information on sensitivity and specificity because the data is heavily dependent on the nature of the definition of periprosthetic infection used and because most classifications have a bias. For example, the sensitivity described in the existing publications varies from 62–100% and the specificity from 64–96% [35,37], with the figures being even lower for low-grade infections with a sensitivity of 66–87% and a specificity of 68–81% [38,39]. Various studies have reported different reference values for serum CRP, with a wide range of values from 3.0–32.0 mg/L (Figure 4). The cut-off value currently used by the EBJIS and ICM is 10 mg/L [9].

Serum fibrinogen is well known for its role in the coagulation cascade and is also closely associated with inflammatory processes. It activates the synthesis of proinflammatory cytokines such as interleukin 6 and tumor necrosis factor alpha and stimulates various immune cells [40]. Various reference values between 432 mg/dL and 519 mg/dL were investigated for fibrinogen [41,42], whereby the optimal cut-off value was 457 mg/dL, which showed a sensitivity of 69% and a specificity of 89% [38].

A variety of cut-off values are also mentioned in various references for erythrocyte sedimentation rate (ICM criteria from 2018 with a cut-off of 30 mm/h), interleukin 6 and procalcitonin, although no optimal values are defined. The combination of different parameters increases the accuracy, but it can in no way prove or rule out a periprosthetic infection.

The different accuracies and threshold values for diagnostics can be explained by a variety of reasons, in particular the different definitions of infection in the individual studies, the heterogeneous group of microorganisms, patient-specific factors (autoimmune diseases, cancer, age, gender, underlying diseases, medication, etc.) and the influence of anti-infective and immunomodulating therapy (corticosteroids) and the length of time after implantation.

In summary, serum biomarkers are an important parameter for obtaining initial indications of a periprosthetic infection and should always be followed up with an extension of the diagnostic work-up in the event of positive findings [38].

### 2.5. Synovial Testing

Joint aspiration under sterile conditions is firmly anchored in the diagnostic algorithm for periprosthetic infection. Historically, in addition to the cell count, pathogen cultivation in particular was used as a diagnostic marker. In addition, synovial biomarkers (alpha-defensin, CRP, leukocyte esterase and interleukin 6) can now be determined, and the fluid can be studied using DNA sequencing (PCR). The accuracy of the individual parameters often depends on the amount of synovial fluid obtained. This can be a limiting factor in hip joint aspiration in particular, as punctio sicca (dry aspiration) often occurs here.

The leukocyte count and the determination of the proportion of polymorphonuclear leukocytes are widely used markers. The sensitivity of the leukocyte count is between 78% and 94%, and the specificity is between 81% and 96%. A sensitivity of 90–97% and a specificity of 84–90% are reported for the percentage of polymorphonuclear leukocytes [43,44,45]. The definition currently used by the EBJIS defines a value of <1500 leukocytes/µL and a fraction of ≤65% polymorphonuclear leukocytes as “infection unlikely” and over 3000 leukocytes/µL and >80% polymorphonuclear leukocytes as “infection confirmed”, while the range in between is defined as “infection likely” [9].

In addition to the quantitative determination of the synovial leukocyte count, some laboratory diagnostic devices (for example, the Yumizen H500, Horiba, Lyon, France) also have the option of creating a graphic cell distribution of existing leukocyte populations. Depending on their volume and light absorption capacity, the respective cell populations are arranged in a coordinate system, and thus a visual representation of their quantitative distribution is possible [46].

The cell volume is determined via an impedance measurement. For this purpose, two electrodes that generate a constant current are arranged around an aperture in a so-called flow cell. When cells flow through the aperture, they generate a certain resistance, the so-called impedance. The potential measured is proportional to the cell size and changes as soon as cells of different sizes flow through the opening. The resulting pulse changes are recorded and analyzed electronically. The cells are then sorted according to size and assigned to a cell population. This volume differentiation is later recorded graphically on the *x*-axis of a cell distribution diagram [46].

The arrangement of the cells along the *Y*-axis, on the other hand, is determined by the contrasting light absorption of the different populations. A so-called electro-optical measurement is carried out in the flow cell, where impedance changes are also measured. Here, non-absorbed scattered light passes through the free spaces in the cell nucleus and thus enables the measurement of optical reactions depending on the respective cell structure and light absorption [46].

**Figure 5:**
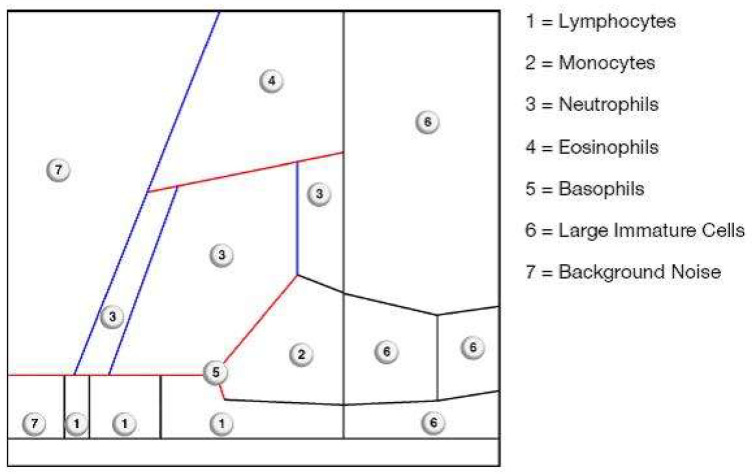
Distribution of the different areas of the LMNE-Matrix.

**Figure 6:**
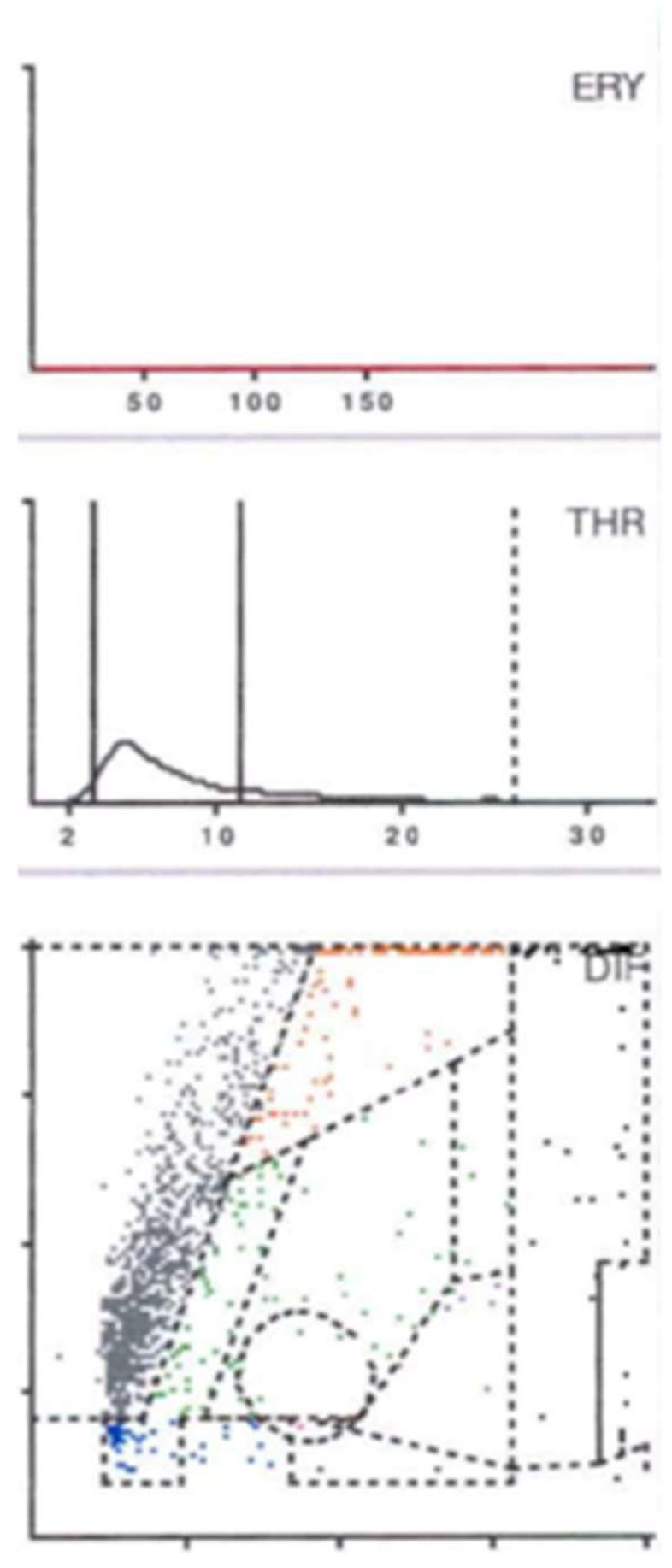
LMNE-Matrix Abraded Type I. 77-year-old woman with aseptic loosening after total hip arthroplasty. Serum CRP 3.1 mg/L; blood white cell count 9300/µL; synovial aspiration cell count 1970/µL, corrected (manual counting) 210/µL, percentage of neutrophils 28.6%; Alpha-Defensin < 0.1.

**Figure 7:**
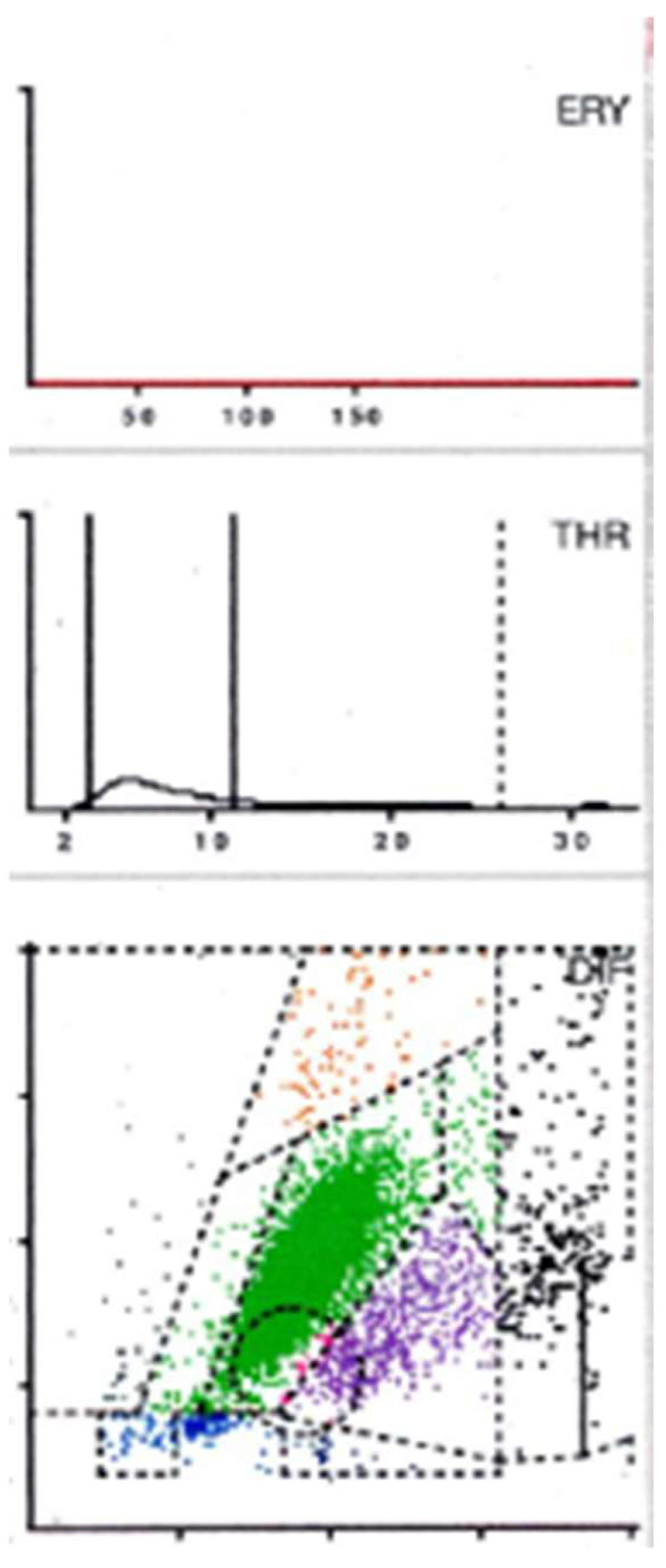
LMNE-Matrix Infection Type II. 58-year-old man with late periprosthetic infection after total hip arthroplasty. Serum CRP 22.2 mg/L; white blood cell count 10,940/µL; synovial aspiration cell count 33,420/µL, corrected (manual counting) 34,460/µL, percentage of neutrophils 93.2%; Alpha-Defensin 3.3.

After each cell has been categorized according to its cell volume and its ability to absorb light along the *X* and *Y* axes, the cell distribution diagram is created in the form of a dot plot (Figure 5, Figure 6 and Figure 7).

Based on the histological classification of periprosthetic tissue published by Morawietz and Krenn [47], the analysis of the aspirate also produces a specific plot that depends on the contents of the synovial fluid. If abraded particles are present in significant numbers, these would be found in the so-called NOISE area and classified as abraded type I (Figure 5). An infected prosthesis, on the other hand, would feature a significant neutrophil area (infection type II) (Figure 6). Both phenomena would be seen in the case of a combined type III of infection and abrasion, while an unclear distribution of cells and particles is found in the indifferent type IV [46].

This subdivision is extremely helpful if the final cell count is subject to the influence of abrasion particles. In the context of automated cell counting, the particles can be incorrectly identified as cells and thus have a false positive influence on the final cell count [48,49].

In contrast, leukocytes can be clearly differentiated from abrasion particles using the graphical representation (so-called LMNE matrix), as these are shown in different fields in each case. Particularly in the presence of metal abrasion particles, the joint aspirate can be macroscopically evaluated as purulent and therefore infected, or may even be accompanied by seemingly high cell counts and elevated CRP and alpha-defensin values. Without the aid of the cell distribution plot, these characteristics could be incorrectly interpreted as a periprosthetic infection [46].

A comparison of the evaluation of the so-called LMNE matrices with the associated histologically examined membrane types classified according to Morawietz and Krenn [47] shows a statistically significant correlation (*p* < 0.001) [46].

If just the two types of infection (type I and type II) are considered in the context of infection diagnostics, the cell count cut-off can also be significantly reduced (1400/μL) without losing sensitivity. As a result, the graphical representation of the cell distribution plot not only provides a reliable diagnostic method but also increases the diagnostic value of synovial cell count determination [46].

Another factor influencing the synovial cell count is the presence of blood in the aspirate. It was recently reported that when the cell distribution diagram (LMNE matrix) showed additional cells in the areas of lymphocytes, basophils and/or eosinophils, a large proportion of the cells shown in the neutrophil leukocyte field were ultimately attributable to the presence of a hematoma [50]. Cell count values in the borderline range can thus be corrected downward and therefore will not be interpreted as an infection. This finding thus helps to differentiate specifically between genuine early periprosthetic infections with elevated leukocyte counts and high cell counts that are due to hemarthrosis [50].

The alpha-defensin test and leukocyte esterase in particular have become established as newer biomarkers. Alpha-defensin is an antimicrobial peptide produced by neutrophil granulocytes. This can be detected using a lateral flow test (10 min) or an ELISA (1–2 days). The sensitivity is reported in the literature as 65–95% and the specificity as 82–100% [51,52,53]. This is comparable to the accuracy of the leukocyte count in combination with the percentage of polymorphonuclear leukocytes and does not necessarily offer any additional diagnostic value [54,55], so the benefits of an additional alpha-defensin test are constantly being questioned. The main advantages are the small amount of synovial fluid required (0.5 mL), the speed of the test result (especially in the lateral flow test) and the independence from prior antibiotic treatment [56]. One disadvantage of the alpha-defensin test is falsification in the case of abrasion-induced metallosis [57] and crystal arthropathies [48].

The detection of leukocyte esterase is a widely available diagnostic tool. It is an enzyme that is secreted by neutrophil granulocytes. As a rapid test, outcomes are available within 10 min, making it suitable for time-limited diagnosis, for example, in the treatment of a periprosthetic fracture. However, the disadvantage is that both abraded particles in metallosis and blood contamination of the aspirate contribute to the falsification of the values [58]. This can be improved by centrifuging the sample beforehand. The sensitivity is given in the literature as 49–95% and the specificity as 82–100% [59,60]. Similar to the alpha-defensin test, prior treatment with antibiotics does not appear to have any influence on the values of leukocyte esterase [61], and the simultaneous determination of alpha-defensin and leukocyte esterase can also lead to an improvement in diagnostic accuracy [62].

As a further diagnostic approach, only systemically available biomarkers such as CRP and interleukin-6 have been determined directly from synovial fluid to date. The intention was that biomarkers from synovial fluid should have a higher specificity. However, the studies carried out on this showed contradictory results; while higher specificity and sensitivity were reported in some cases [63,64], this could not be confirmed in others [65].

In particular, the combination of CRP, synovial CRP and alpha-defensin appears to increase sensitivity by up to 97% and specificity by up to 100% [66,67,68].

Interleukin 6 is an inflammatory cytokine and is currently one of the most interesting areas of research, as it has shown a high degree of accuracy in terms of diagnostics even under active antibiotic treatment. Further studies are currently being conducted, but initial results have shown promising approaches with sensitivity and specificity of up to 100% [69,70,71].

Neutrophil gelatinase-associated lipocalin (NGAL) [72,73] and lactoferrin [74] also show promising values for sensitivity and specificity as new biomarkers used in PJI diagnostics, so it will be important for future research to establish precise cut-off values for these markers in cases of PJI.

Synovial contamination with blood or saline has an important influence on cytological findings. The risk of blood contamination is particularly increased if the examiner misses the joint capsule and thus aspirates blood from the surrounding tissue or if there is a hemarthrosis. In the case of a punctio sicca (dry aspiration), some examiners use an injection of saline solution to flush the joint and thus obtain sufficient fluid. However, these admixtures can reduce the concentration of biomarkers and cells, rendering established limits invalid. Compared to aspirates without admixtures, this demonstrably leads to a reduction in sensitivity. This loss of sensitivity was illustrated using the examples of cell count (93% to 69%), PMN% (95% to 88%), CRP (88% to 65%) and α-defensin (93% to 70%) [75]. This considerably relativizes the diagnostic significance of the individual values, so results from diluted aspirates should be excluded and not used for diagnosis [76].

### 2.6. Multiplex Polymerase Chain Reaction (mPCR)

mPCR is a method that offers promising approaches for improving the diagnosis of periprosthetic infection. The genotypes of the bacteria are determined by detecting DNA. In addition to the small amount of liquid required (180 µL, e.g., aspirate or sonication medium) [77], the advantages of the method are the rapid acquisition of results (approximately 5–7 h) and the potential availability of data relating to pathogens and even resistance. However, PCR does not provide any information as to whether it is a past infection that has led to the presence of bacterial DNA or whether there is an active periprosthetic infection. This results in a higher proportion of false-positive test results and explains the sometimes low sensitivity (75–85%) and high specificity (94–98%) [52,78]. This is precisely why the combination of PCR with “classical diagnostic criteria” makes sense, as significantly higher accuracies can be achieved with this approach [77,79].

In particular, the amount of microbial DNA per tissue sample plays a decisive role in making precise statements about the pathogens and the antibiotic resistance of the pathogens. Because of the often small amounts of DNA, this is not always possible, so there is no added diagnostic value [80]. Furthermore, pathogens for which no specific primer is yet available within the mPCR starter cassette cannot be detected.

### 2.7. Next-Generation Sequencing

Next-generation sequencing (NGS) offers another option for studying DNA. In contrast to PCR, no primers are used to obtain information by amplifying an existing gene. Instead, existing DNA fragments are “broken down”, amplified en masse and later assembled in so-called “reads” and compared with a database. Due to the massive improvement in data throughput, the accuracy of the method has continued to increase in recent years [81]. A sensitivity of approx. 90% is reported [82], while the specificity is somewhat lower in some cases. However, this may be due to the high risk of contamination and the corresponding rate of false-positive outcomes [83,84].

In summary, it can be said that NGS is currently not yet superior to classical cultivation in terms of accuracy [85]. However, it offers a promising option for the future, especially for the detection of previously culture-negative infections [86], whereby it should be used above all when the biomarkers indicate a very high probability of an infection and no pathogen has yet been detected [87].

### 2.8. Microbiology

The microbial cultivation of pathogens is an important tool in the diagnosis of periprosthetic infections and is particularly essential for treatment planning and perioperative antibiotic administration. It should therefore be part of every PJI diagnosis [88]. However, as a single test, direct pathogen detection is of lesser importance because of its sometimes suboptimal sensitivity [89,90,91].

The influence of previous antibiotic therapy, the difficulty of cultivating slow-growing microorganisms and the risk of contamination are of particular importance [92]. In addition to synovial fluid, periprosthetic tissue and prosthesis components, which are removed during revision surgery and then processed using sonication, are used as sample material. During sonication, biofilms are gently removed from the surface of the prosthesis into liquid using ultrasound. Sonication appears to be particularly important in culture-negative cases and with prior administration of antibiotics, while overall it is inferior to the accuracy of culturing periprosthetic tissue in terms of sensitivity and specificity [93]. An infection must be assumed whenever bacteria are recovered from the sonication material, and an infection is considered confirmed for unenriched samples that result in a density of >50 colony-forming units/mL [92,94].

The diagnostic collection of tissue samples using biopsy forceps represents a valuable additional diagnostic procedure, especially in the case of punctio sicca (dry aspiration). When obtaining tissue samples, at least 3–5 samples should be obtained, which should be transferred, together with the aspirates, to the microbiology laboratory as soon as possible [95] and incubated there for 14 days (like the aspirate) [9,96]. The incubation media must be able to support bacteria with low metabolic activity, polymicrobial infections and almost non-viable bacteria (e.g., due to antibiosis); chocolate agar, MacConkey agar, thioglycolate broth, etc. are used for this purpose [97].

Samples should be obtained from periarticular membranes and synovia, while accuracy is highest at the bone/prosthesis interface [98,99,100].

If possible, a 14-day antibiotic-free interval is desirable before obtaining samples [101,102], although preoperative antibiotic prophylaxis does not appear to have any influence on the identification of pathogens and should therefore be administered if deemed necessary [103,104].

Two phenotypically identical pathogens obtained from two different samples are considered to be evidence of infection [95]. One positive sample can only be evaluated in the overall context of the other findings. Virulent pathogens and pathogens unusual for contamination (e.g., Staph. aureus or Gram-negative rods) are more likely to indicate an infection than typical pathogens of the skin flora (e.g., coagulase-negative staphylococci or Cutibacterium acnes), but in both cases, further diagnostics should always be carried out promptly.

### 2.9. Histology

Histology can detect cytological changes caused by inflammatory reactions. This involves the infiltration of leukocytes into the surrounding tissue, and this can be detected under the microscope. Morawietz and Krenn have developed a histological classification that distinguishes between four types of periprosthetic membrane and synovium: abrasion type I, infection type II, mixed type (infection and abrasion) III and indifferent type IV [47].

Furthermore, microorganisms can sometimes be detected directly; although this is rare, it has a very high specificity [97,105]. Tissue samples from at least three (or better, five) different locations should be studied, preferably obtained from the prosthesis/bone interface, the synovium/pseudocapsule and from abnormally altered tissue. A value of >5 neutrophil granulocytes (PMN) in at least five High Power Fields (HPF with ×400 magnification) is the current reference value [9,98], which corresponds to the value already recommended in 1976 [106]. The sensitivity and specificity depend on the defined cut-off value. For a cut-off value of 5 PMN, for example, a sensitivity of approx. 93% and a specificity of 84% were reported; while the specificity increases with higher cut-off values, the sensitivity decreases [107,108].

False positive values can occur in the context of fractures, for example, because in such cases, inflammatory cells also infiltrate the damaged tissue. In some cases, lower leukocyte counts/HPF have also been reported, so immunohistochemical studies can probably contribute to a more accurate diagnosis in the future [109].

Histopathological examination also represents an important diagnostic tool prior to revision surgery and can be used as part of the diagnostic process when the outcome of serological and synovial diagnostics is unclear [110]. Biopsy therefore represents an important diagnostic method, especially in the case of punctio sicca (dry aspiration), as it has the advantage that the periprosthetic tissue obtained can be used for cultivation as well as for histological assessment. In addition, during this diagnostic procedure under anesthesia, synovia can usually be obtained for diagnostic synovial analysis. The concurrent use of different diagnostic methods involving periprosthetic tissue and synovia increases the accuracy of the diagnostic biopsy procedure to 98% in the knee joint and 93% in the hip joint [110,111].

## 3. Summary

**Figure 8:**
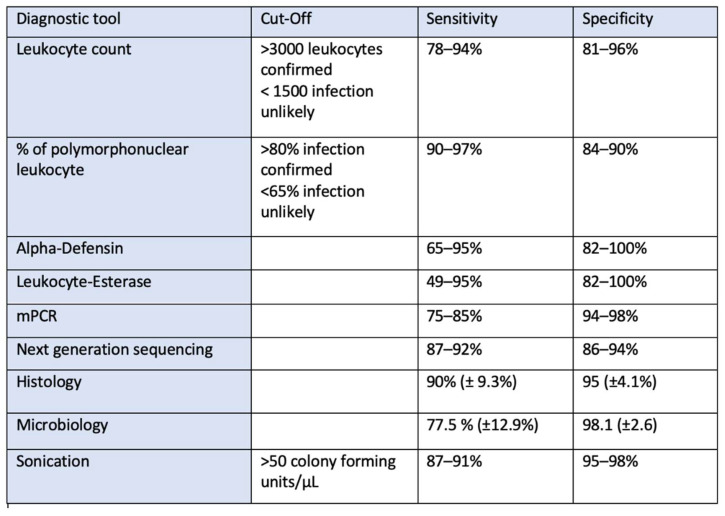
Overview of the different diagnostic tools with sensitivity and specificity.

The diagnostic methods and tests listed above show that a combination of different tests increases the accuracy of the diagnosis. The specificity and sensitivity for the diagnostic tests are listed in Figure 8. Each physician or clinical department must select the tests from the available methods that can be best implemented for them in organizational and technical terms.

Simple serological parameters such as CRP (because of its technical simplicity) and the aspiration of the joint with the cultivation of the aspirate (because of the importance of pathogen isolation) should always be included. Which additional synovial tests are carried out certainly also depends on the amount of aspirate that is obtained. In the case of ambiguous findings or punctio sicca (dry aspiration), diagnostic tissue biopsy is recommended, as it offers the possibility of culturing several tissue samples and performing additional histological studies [88,112].

**Figure 9:**
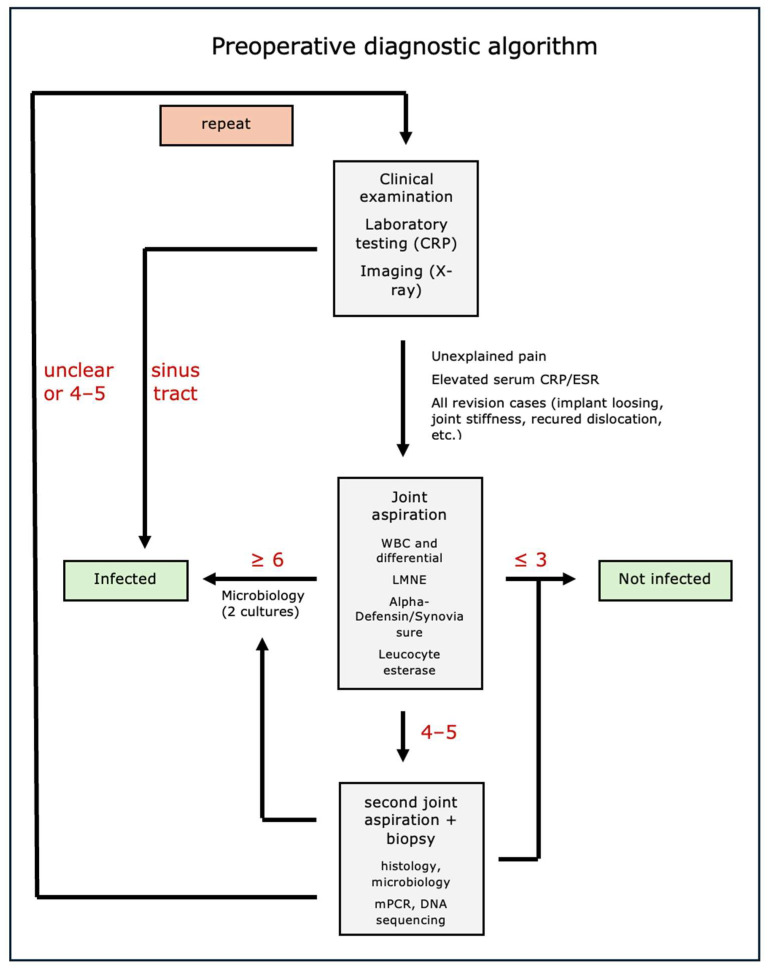
Preoperative diagnostic algorithm aligned with the definition of ICM.

As a result, our clinic has developed a treatment algorithm closely aligned with the definition of ICM, as depicted in Figure 9. Following the preceding clinical examination and serum diagnostics, the synovial examination is particularly crucial, with biopsy being the central component in cases with unclear findings. Further diagnostic procedures involving imaging and genetic techniques are especially valuable in specific cases (e.g., during ongoing antibiotic therapy) for determining pathogens and antibiotic resistance.

## Data Availability

The data presented in this study are available on request from the corresponding author. The data are not publicly available due to privacy.

## References

[1] Rupp M., Lau E., Kurtz S.M., Alt V. (2020). Projections of Primary TKA and THA in Germany from 2016 through 2040. Clin. Orthop. Relat. Res..

[2] Slifka K.J., Yi S.H., Reddy S.C., Baggs J., Jernigan J.A. (2018). 287. The Attributable Mortality of Prosthetic Joint Infection After Primary Hip and Knee Arthroplasty Among Medicare Beneficiaries, 2005–2012. Open Forum Infect. Dis..

[3] Coventry M.B. (1975). Treatment of Infections Occurring in Total Hip Surgery. Orthop. Clin. N. Am..

[4] Tsukayama D.T., Estrada R., Gustilo R.B. (1996). Infection after Total Hip Arthroplasty. A Study of the Treatment of One Hundred and Six Infections. J. Bone Jt. Surg. Am..

[5] Tornero E., Soriano A. (2016). Importance of Selection and Duration of Antibiotic Regimen in Prosthetic Joint Infections Treated with Debridement and Implant Retention—Authors’ Response. J. Antimicrob. Chemother..

[6] Deckey D.G., Christopher Z.K., Bingham J.S., Spangehl M.J. (2023). Principles of Mechanical and Chemical Debridement with Implant Retention. Arthroplasty.

[7] van der Ende B., van Oldenrijk J., Reijman M., Croughs P.D., van Steenbergen L.N., Verhaar J.A.N., Bos P.K. (2021). Timing of Debridement, Antibiotics, and Implant Retention (DAIR) for Early Post-Surgical Hip and Knee Prosthetic Joint Infection (PJI) Does Not Affect 1-Year Re-Revision Rates: Data from the Dutch Arthroplasty Register. J. Bone Jt. Infect..

[8] Li C., Ojeda-Thies C., Renz N., Margaryan D., Perka C., Trampuz A. (2020). The Global State of Clinical Research and Trends in Periprosthetic Joint Infection: A Bibliometric Analysis. Int. J. Infect. Dis..

[9] McNally M., Sousa R., Wouthuyzen-Bakker M., Chen A.F., Soriano A., Vogely H.C., Clauss M., Higuera C.A., Trebše R. (2021). The EBJIS Definition of Periprosthetic Joint Infection. Bone Jt. J..

[10] Fink B., Schlumberger M., Beyersdorff J., Schuster P. (2020). C-Reactive Protein Is Not a Screening Tool for Late Periprosthetic Joint Infection. J. Orthop. Traumatol..

[11] Kim S.-J., Cho Y.J. (2021). Current Guideline for Diagnosis of Periprosthetic Joint Infection: A Review Article. Hip Pelvis.

[12] McNally M., Sigmund I., Hotchen A., Sousa R. (2023). Making the Diagnosis in Prosthetic Joint Infection: A European View. EFORT Open Rev..

[13] Osmon D.R., Berbari E.F., Berendt A.R., Lew D., Zimmerli W., Steckelberg J.M., Rao N., Hanssen A., Wilson W.R. (2013). Infectious Diseases Society of America Diagnosis and Management of Prosthetic Joint Infection: Clinical Practice Guidelines by the Infectious Diseases Society of America. Clin. Infect. Dis..

[14] Parvizi J., Gehrke T. (2014). International Consensus Group on Periprosthetic Joint Infection Definition of Periprosthetic Joint Infection. J. Arthroplast..

[15] Parvizi J., Tan T.L., Goswami K., Higuera C., Della Valle C., Chen A.F., Shohat N. (2018). The 2018 Definition of Periprosthetic Hip and Knee Infection: An Evidence-Based and Validated Criteria. J. Arthroplast..

[16] Izakovicova P., Borens O., Trampuz A. (2019). Periprosthetic Joint Infection: Current Concepts and Outlook. EFORT Open Rev..

[17] Shohat N., Goswami K., Tan T.L., Henstenburg B., Makar G., Rondon A.J., Parvizi J. (2019). Fever and Erythema Are Specific Findings in Detecting Infection Following Total Knee Arthroplasty. J. Bone Jt. Infect..

[18] Zahar A., Sarungi M. (2021). Diagnosis and Management of the Infected Total Knee Replacement: A Practical Surgical Guide. J. Exp. Orthop..

[19] Zajonz D., Wuthe L., Tiepolt S., Brandmeier P., Prietzel T., von Salis-Soglio G.F., Roth A., Josten C., Heyde C.-E., Ghanem M. (2015). Diagnostic Work-up Strategy for Periprosthetic Joint Infections after Total Hip and Knee Arthroplasty: A 12-Year Experience on 320 Consecutive Cases. Patient Saf. Surg..

[20] Porrino J., Wang A., Moats A., Mulcahy H., Kani K. (2020). Prosthetic Joint Infections: Diagnosis, Management, and Complications of the Two-Stage Replacement Arthroplasty. Skeletal Radiol..

[21] Saeed K. (2014). Diagnostics in Prosthetic Joint Infections. J. Antimicrob. Chemother..

[22] Otto-Lambertz C., Yagdiran A., Wallscheid F., Eysel P., Jung N. (2017). Periprosthetic Infection in Joint Replacement. Dtsch. Arztebl. Int..

[23] Signore A., Sconfienza L.M., Borens O., Glaudemans A.W.J.M., Cassar-Pullicino V., Trampuz A., Winkler H., Gheysens O., Vanhoenacker F.M.H.M., Petrosillo N. (2019). Consensus Document for the Diagnosis of Prosthetic Joint Infections: A Joint Paper by the EANM, EBJIS, and ESR (with ESCMID Endorsement). Eur. J. Nucl. Med. Mol. Imaging.

[24] Talbot B.S., Weinberg E.P. (2016). MR Imaging with Metal-Suppression Sequences for Evaluation of Total Joint Arthroplasty. Radiographics.

[25] Shufen C., Jinmin L., Xiaohui Z., Bin G. (2023). Diagnostic Value of Magnetic Resonance Imaging for Patients with Periprosthetic Joint Infection: A Systematic Review. BMC Musculoskelet. Disord..

[26] Sconfienza L.M., Signore A., Cassar-Pullicino V., Cataldo M.A., Gheysens O., Borens O., Trampuz A., Wörtler K., Petrosillo N., Winkler H. (2019). Diagnosis of Peripheral Bone and Prosthetic Joint Infections: Overview on the Consensus Documents by the EANM, EBJIS, and ESR (with ESCMID Endorsement). Eur. Radiol..

[27] Glaudemans A.W.J.M., de Vries E.F.J., Vermeulen L.E.M., Slart R.H.J.A., Dierckx R.A.J.O., Signore A. (2013). A Large Retrospective Single-Centre Study to Define the Best Image Acquisition Protocols and Interpretation Criteria for White Blood Cell Scintigraphy with 99mTc-HMPAO-Labelled Leucocytes in Musculoskeletal Infections. Eur. J. Nucl. Med. Mol. Imaging.

[28] Ottink K.D., Gelderman S.J., Wouthuyzen-Bakker M., Ploegmakers J.J.W., Glaudemans A.W.J.M., Jutte P.C. (2022). Nuclear Imaging Does Not Have Clear Added Value in Patients with Low a Priori Chance of Periprosthetic Joint Infection. A Retrospective Single-Center Experience. J. Bone Jt. Infect..

[29] Verberne S.J., Raijmakers P.G., Temmerman O.P.P. (2016). The Accuracy of Imaging Techniques in the Assessment of Periprosthetic Hip Infection: A Systematic Review and Meta-Analysis. J. Bone Jt. Surg. Am..

[30] Wang Y., Wang R., Geng L., Li Q., Qi E., Shi Y., Wang Y., Zheng Q., Zhang G., Chen J. (2022). Different Uptake Patterns of 68Ga-FAPI in Aseptic Loosening and Periprosthetic Joint Infection of Hip Arthroplasty: A Case Series and Literature Review. Front. Med..

[31] Buechler M.B., Fu W., Turley S.J. (2021). Fibroblast-Macrophage Reciprocal Interactions in Health, Fibrosis, and Cancer. Immunity.

[32] Wang Y., Wang R., Zhang X., Li L., Liu H., Chang Y., Li Q., Wang Y., Qi E., Hao L. (2023). Diagnostic Efficiency of [68 Ga]Ga-DOTA-FAPI-04 in Differentiating Periprosthetic Hip Joint Infection and Aseptic Failure. Eur. J. Nucl. Med. Mol. Imaging.

[33] Welling M.M., Warbroek K., Khurshid C., van Oosterom M.N., Rietbergen D.D.D., de Boer M.G.J., Nelissen R.G.H.H., van Leeuwen F.W.B., Pijls B.G., Buckle T. (2023). A Radio- and Fluorescently Labelled Tracer for Imaging and Quantification of Bacterial Infection on Orthopaedic Prostheses. Bone Jt. Res..

[34] Sigmund I.K., Dudareva M., Watts D., Morgenstern M., Athanasou N.A., McNally M.A. (2020). Limited Diagnostic Value of Serum Inflammatory Biomarkers in the Diagnosis of Fracture-Related Infections. Bone Jt. J..

[35] Sigmund I.K., Puchner S.E., Windhager R. (2021). Serum Inflammatory Biomarkers in the Diagnosis of Periprosthetic Joint Infections. Biomedicines.

[36] Berbari E., Mabry T., Tsaras G., Spangehl M., Erwin P.J., Murad M.H., Steckelberg J., Osmon D. (2010). Inflammatory Blood Laboratory Levels as Markers of Prosthetic Joint Infection: A Systematic Review and Meta-Analysis. J. Bone Jt. Surg. Am..

[37] Qin L., Li F., Gong X., Wang J., Huang W., Hu N. (2020). Combined Measurement of D-Dimer and C-Reactive Protein Levels: Highly Accurate for Diagnosing Chronic Periprosthetic Joint Infection. J. Arthroplast..

[38] Sigmund I.K., Holinka J., Staats K., Sevelda F., Lass R., Kubista B., Giurea A., Windhager R. (2021). Inferior Performance of Established and Novel Serum Inflammatory Markers in Diagnosing Periprosthetic Joint Infections. Int. Orthop..

[39] Akgün D., Müller M., Perka C., Winkler T. (2018). The Serum Level of C-Reactive Protein Alone Cannot Be Used for the Diagnosis of Prosthetic Joint Infections, Especially in Those Caused by Organisms of Low Virulence. Bone Jt. J..

[40] Adams R.A., Passino M., Sachs B.D., Nuriel T., Akassoglou K. (2004). Fibrin Mechanisms and Functions in Nervous System Pathology. Mol. Interv..

[41] Klim S.M., Amerstorfer F., Gruber G., Bernhardt G.A., Radl R., Leitner L., Leithner A., Glehr M. (2018). Fibrinogen—A Practical and Cost Efficient Biomarker for Detecting Periprosthetic Joint Infection. Sci. Rep..

[42] Alturfan A.A., Eralp L., Emekli N. (2008). Investigation of Inflammatory and Hemostatic Parameters in Female Patients Undergoing Total Knee Arthroplasty Surgery. Inflammation.

[43] Levent A., Neufeld M.E., Piakong P., Lausmann C., Gehrke T., Citak M. (2021). Which International Consensus Meeting Preoperative Minor Criteria Is the Most Accurate Marker for the Diagnosis of Periprosthetic Joint Infection in Hip and Knee Arthroplasty?. J. Arthroplast..

[44] Ivy M.I., Sharma K., Greenwood-Quaintance K.E., Tande A.J., Osmon D.R., Berbari E.F., Mandrekar J., Beauchamp C.P., Hanssen A.D., Abdel M.P. (2021). Synovial Fluid α Defensin Has Comparable Accuracy to Synovial Fluid White Blood Cell Count and Polymorphonuclear Percentage for Periprosthetic Joint Infection Diagnosis. Bone Jt. J..

[45] Tang H., Xu J., Yuan W., Wang Y., Yue B., Qu X. (2022). Reliable Diagnostic Tests and Thresholds for Preoperative Diagnosis of Non-Inflammatory Arthritis Periprosthetic Joint Infection: A Meta-Analysis and Systematic Review. Orthop. Surg..

[46] Fink B., Hoyka M., Weissbarth E., Schuster P., Berger I. (2021). The Graphical Representation of Cell Count Representation: A New Procedure for the Diagnosis of Periprosthetic Joint Infections. Antibiotics.

[47] Krenn V., Morawietz L., Jakobs M., Kienapfel H., Ascherl R., Bause L., Kuhn H., Matziolis G., Skutek M., Gehrke T. (2011). Joint endoprosthesis pathology. Histopathological diagnostics and classification. Pathologe.

[48] Partridge D.G., Gordon A., Townsend R. (2017). False-Positive Synovial Fluid Alpha-Defensin Test in a Patient with Acute Gout Affecting a Prosthetic Knee. Eur. J. Orthop. Surg. Traumatol..

[49] Deirmengian C.A., Kazarian G.S., Feeley S.P., Sizer S.C. (2020). False-Positive Automated Synovial Fluid White Blood Cell Counting Is a Concern for Both Hip and Knee Arthroplasty Aspirates. J. Arthroplast..

[50] Fink B., Hoyka M., Weissbarth E., Schuster P., Berger I. (2022). A New Graphic Type Differentiation of Cell Account Determination for Distinguishing Acute Periprosthetic Joint Infection from Hemarthrosis. Antibiotics.

[51] Aalirezaie A., Bauer T.W., Fayaz H., Griffin W., Higuera C.A., Krenn V., Krenn V., Molano M., Moojen D.-J., Restrepo C. (2019). Hip and Knee Section, Diagnosis, Reimplantation: Proceedings of International Consensus on Orthopedic Infections. J. Arthroplast..

[52] Lausmann C., Kolle K.N., Citak M., Abdelaziz H., Schulmeyer J., Delgado G.D., Gehrke T., Gebauer M., Zahar A. (2020). How Reliable Is the next Generation of Multiplex-PCR for Diagnosing Prosthetic Joint Infection Compared to the MSIS Criteria? Still Missing the Ideal Test. Hip Int..

[53] Kuiper J.W.P., Verberne S.J., Vos S.J., van Egmond P.W. (2020). Does the Alpha Defensin ELISA Test Perform Better than the Alpha Defensin Lateral Flow Test for PJI Diagnosis? A Systematic Review and Meta-Analysis of Prospective Studies. Clin. Orthop. Relat. Res..

[54] Kleeman-Forsthuber L.T., Dennis D.A., Brady A.C., Pollet A.K., Johnson R.M., Jennings J.M. (2021). Alpha-Defensin Is Not Superior to Traditional Diagnostic Methods for Detection of Periprosthetic Joint Infection in Total Hip Arthroplasty and Total Knee Arthroplasty Patients. J. Arthroplast..

[55] Owens J.M., Dennis D.A., Abila P.M., Johnson R.M., Jennings J.M. (2022). Alpha-Defensin Offers Limited Utility in Work-Up Prior to Reimplantation in Chronic Periprosthetic Joint Infection in Total Joint Arthroplasty Patients. J. Arthroplast..

[56] Shahi A., Parvizi J., Kazarian G.S., Higuera C., Frangiamore S., Bingham J., Beauchamp C., Valle C.D., Deirmengian C. (2016). The Alpha-Defensin Test for Periprosthetic Joint Infections Is Not Affected by Prior Antibiotic Administration. Clin. Orthop. Relat. Res..

[57] Okroj K.T., Calkins T.E., Kayupov E., Kheir M.M., Bingham J.S., Beauchamp C.P., Parvizi J., Della Valle C.J. (2018). The Alpha-Defensin Test for Diagnosing Periprosthetic Joint Infection in the Setting of an Adverse Local Tissue Reaction Secondary to a Failed Metal-on-Metal Bearing or Corrosion at the Head-Neck Junction. J. Arthroplast..

[58] McNabb D.C., Dennis D.A., Kim R.H., Miner T.M., Yang C.C., Jennings J.M. (2017). Determining False Positive Rates of Leukocyte Esterase Reagent Strip When Used as a Detection Tool for Joint Infection. J. Arthroplast..

[59] Wyatt M.C., Beswick A.D., Kunutsor S.K., Wilson M.J., Whitehouse M.R., Blom A.W. (2016). The Alpha-Defensin Immunoassay and Leukocyte Esterase Colorimetric Strip Test for the Diagnosis of Periprosthetic Infection. J. Bone Jt. Surg. Am..

[60] Yu B.-Z., Li R., Fu J., Chai W., Hao L.-B., Chen J.-Y. (2021). Leukocyte Esterase Test and Alpha-Defensin Test Have Similar Accuracy for the Diagnosis of Periprosthetic Joint Infection. Int. Orthop..

[61] Shahi A., Alvand A., Ghanem E., Restrepo C., Parvizi J. (2019). The Leukocyte Esterase Test for Periprosthetic Joint Infection Is Not Affected by Prior Antibiotic Administration. J. Bone Jt. Surg. Am..

[62] Grünwald L., Schmidutz F., Döttger P., Erne F., Schreiner A.J., Hemmann P. (2023). Leukocyte Esterase and Alpha-Defensin in Periprosthetic Joint Infection: Predictive Quality and Correlation in a Prospective Study. Int. Orthop..

[63] Omar M., Ettinger M., Reichling M., Petri M., Guenther D., Gehrke T., Krettek C., Mommsen P. (2015). Synovial C-Reactive Protein as a Marker for Chronic Periprosthetic Infection in Total Hip Arthroplasty. Bone Jt. J..

[64] Parvizi J., Jacovides C., Adeli B., Jung K.A., Hozack W.J., Mark B. (2012). Coventry Award: Synovial C-Reactive Protein: A Prospective Evaluation of a Molecular Marker for Periprosthetic Knee Joint Infection. Clin. Orthop. Relat. Res..

[65] Tetreault M.W., Wetters N.G., Moric M., Gross C.E., Della Valle C.J. (2014). Is Synovial C-Reactive Protein a Useful Marker for Periprosthetic Joint Infection?. Clin. Orthop. Relat. Res..

[66] Deirmengian C., Kardos K., Kilmartin P., Cameron A., Schiller K., Parvizi J. (2014). Combined Measurement of Synovial Fluid α-Defensin and C-Reactive Protein Levels: Highly Accurate for Diagnosing Periprosthetic Joint Infection. J. Bone Jt. Surg. Am..

[67] Baker C.M., Goh G.S., Tarabichi S., Shohat N., Parvizi J. (2022). Synovial C-Reactive Protein Is a Useful Adjunct for Diagnosis of Periprosthetic Joint Infection. J. Arthroplast..

[68] Wang H., Qin L., Wang J., Hu N., Huang W. (2021). Combined Serum and Synovial C-Reactive Protein Tests: A Valuable Adjunct to the Diagnosis of Chronic Prosthetic Joint Infection. BMC Musculoskelet. Disord..

[69] Yu B.-Z., Li R., Li X., Chai W., Zhou Y.-G., Chen J.-Y. (2021). The Relationship of C-Reactive Protein/Interleukin-6 Concentrations between Serum and Synovial Fluid in the Diagnosis of Periprosthetic Joint Infection. J. Orthop. Surg. Res..

[70] Gallo J., Svoboda M., Zapletalova J., Proskova J., Juranova J. (2018). Serum IL-6 in Combination with Synovial IL-6/CRP Shows Excellent Diagnostic Power to Detect Hip and Knee Prosthetic Joint Infection. PLoS ONE.

[71] Randau T.M., Friedrich M.J., Wimmer M.D., Reichert B., Kuberra D., Stoffel-Wagner B., Limmer A., Wirtz D.C., Gravius S. (2014). Interleukin-6 in Serum and in Synovial Fluid Enhances the Differentiation between Periprosthetic Joint Infection and Aseptic Loosening. PLoS ONE.

[72] Huang Z., Zhang Z., Li M., Li W., Fang X., Zhang W. (2022). Synovial Fluid Neutrophil Gelatinase-Associated Lipocalin Can Be Used to Accurately Diagnose Prosthetic Joint Infection. Int. J. Infect. Dis..

[73] Dijkman C., Thomas A.R., Koenraadt K.L.M., Ermens A.a.M., van Geenen R.C.I. (2020). Synovial Neutrophilic Gelatinase-Associated Lipocalin in the Diagnosis of Periprosthetic Joint Infection after Total Knee Arthroplasty. Arch. Orthop. Trauma. Surg..

[74] Wang C., Wang Q., Li R., Qin J., Song L., Zhang Q., Liu M., Chen J., Wang C. (2019). LTF, PRTN3, and MNDA in Synovial Fluid as Promising Biomarkers for Periprosthetic Joint Infection: Identification by Quadrupole Orbital-Trap Mass Spectrometry. J. Bone Jt. Surg. Am..

[75] Deirmengian C., Kardos K., Kilmartin P., Cameron A., Schiller K., Parvizi J. (2014). Diagnosing Periprosthetic Joint Infection: Has the Era of the Biomarker Arrived?. Clin. Orthop. Relat. Res..

[76] Deirmengian C., Feeley S., Kazarian G.S., Kardos K. (2020). Synovial Fluid Aspirates Diluted with Saline or Blood Reduce the Sensitivity of Traditional and Contemporary Synovial Fluid Biomarkers. Clin. Orthop. Relat. Res..

[77] Sigmund I.K., Windhager R., Sevelda F., Staats K., Puchner S.E., Stenicka S., Thalhammer F., Holinka J. (2019). Multiplex PCR Unyvero I60 ITI Application Improves Detection of Low-Virulent Microorganisms in Periprosthetic Joint Infections. Int. Orthop..

[78] Meta-Analysis of Sonication Prosthetic Fluid PCR for Diagnosing Periprosthetic Joint Infection—PMC. https://www.ncbi.nlm.nih.gov/pmc/articles/PMC5922553/.

[79] Suren C., Feihl S., Cabric S., Banke I.J., Haller B., Trampuz A., von Eisenhart-Rothe R., Prodinger P.M. (2020). Improved Pre-Operative Diagnostic Accuracy for Low-Grade Prosthetic Joint Infections Using Second-Generation Multiplex Polymerase Chain Reaction on Joint Fluid Aspirate. Int. Orthop..

[80] Sigmund I.K., Renz N., Feihl S., Morgenstern C., Cabric S., Trampuz A. (2020). Value of Multiplex PCR for Detection of Antimicrobial Resistance in Samples Retrieved from Patients with Orthopaedic Infections. BMC Microbiol..

[81] Mei J., Hu H., Zhu S., Ding H., Huang Z., Li W., Yang B., Zhang W., Fang X. (2023). Diagnostic Role of mNGS in Polymicrobial Periprosthetic Joint Infection. J. Clin. Med..

[82] Wang C., Huang Z., Li W., Fang X., Zhang W. (2020). Can Metagenomic Next-Generation Sequencing Identify the Pathogens Responsible for Culture-Negative Prosthetic Joint Infection?. BMC Infect. Dis..

[83] Ivy M.I., Thoendel M.J., Jeraldo P.R., Greenwood-Quaintance K.E., Hanssen A.D., Abdel M.P., Chia N., Yao J.Z., Tande A.J., Mandrekar J.N. (2018). Direct Detection and Identification of Prosthetic Joint Infection Pathogens in Synovial Fluid by Metagenomic Shotgun Sequencing. J. Clin. Microbiol..

[84] Street T.L., Sanderson N.D., Atkins B.L., Brent A.J., Cole K., Foster D., McNally M.A., Oakley S., Peto L., Taylor A. (2017). Molecular Diagnosis of Orthopedic-Device-Related Infection Directly from Sonication Fluid by Metagenomic Sequencing. J. Clin. Microbiol..

[85] Kildow B.J., Ryan S.P., Danilkowicz R., Lazarides A.L., Penrose C., Bolognesi M.P., Jiranek W., Seyler T.M. (2021). Next-Generation Sequencing Not Superior to Culture in Periprosthetic Joint Infection Diagnosis. Bone Jt. J..

[86] Goswami K., Clarkson S., Phillips C.D., Dennis D.A., Klatt B.A., O’Malley M.J., Smith E.L., Gililland J.M., Pelt C.E., Peters C.L. (2022). An Enhanced Understanding of Culture-Negative Periprosthetic Joint Infection with Next-Generation Sequencing: A Multicenter Study. J. Bone Jt. Surg. Am..

[87] Torchia M.T., Austin D.C., Kunkel S.T., Dwyer K.W., Moschetti W.E. (2019). Next-Generation Sequencing vs Culture-Based Methods for Diagnosing Periprosthetic Joint Infection After Total Knee Arthroplasty: A Cost-Effectiveness Analysis. J. Arthroplast..

[88] Fink B., Lass R. (2016). Diagnostic Algorithm for Failure Analysis of Painful Total Hip Arthroplasties. Z. Orthop. Unf..

[89] Qu X., Zhai Z., Wu C., Jin F., Li H., Wang L., Liu G., Liu X., Wang W., Li H. (2013). Preoperative Aspiration Culture for Preoperative Diagnosis of Infection in Total Hip or Knee Arthroplasty. J. Clin. Microbiol..

[90] Ali F., Wilkinson J.M., Cooper J.R., Kerry R.M., Hamer A.J., Norman P., Stockley I. (2006). Accuracy of Joint Aspiration for the Preoperative Diagnosis of Infection in Total Hip Arthroplasty. J. Arthroplast..

[91] Berbari E.F., Marculescu C., Sia I., Lahr B.D., Hanssen A.D., Steckelberg J.M., Gullerud R., Osmon D.R. (2007). Culture-Negative Prosthetic Joint Infection. Clin. Infect. Dis..

[92] Trampuz A., Piper K.E., Jacobson M.J., Hanssen A.D., Unni K.K., Osmon D.R., Mandrekar J.N., Cockerill F.R., Steckelberg J.M., Greenleaf J.F. (2007). Sonication of Removed Hip and Knee Prostheses for Diagnosis of Infection. N. Engl. J. Med..

[93] Peel T.N., Spelman T., Dylla B.L., Hughes J.G., Greenwood-Quaintance K.E., Cheng A.C., Mandrekar J.N., Patel R. (2017). Optimal Periprosthetic Tissue Specimen Number for Diagnosis of Prosthetic Joint Infection. J. Clin. Microbiol..

[94] Holinka J., Bauer L., Hirschl A.M., Graninger W., Windhager R., Presterl E. (2011). Sonication Cultures of Explanted Components as an Add-on Test to Routinely Conducted Microbiological Diagnostics Improve Pathogen Detection. J. Orthop. Res..

[95] Atkins B.L., Athanasou N., Deeks J.J., Crook D.W.M., Simpson H., Peto T.E.A., McLardy-Smith P., Berendt A.R., Group T.O.C.S. (1998). Prospective Evaluation of Criteria for Microbiological Diagnosis of Prosthetic-Joint Infection at Revision Arthroplasty. J. Clin. Microbiol..

[96] Schäfer P., Fink B., Sandow D., Margull A., Berger I., Frommelt L. (2008). Prolonged Bacterial Culture to Identify Late Periprosthetic Joint Infection: A Promising Strategy. Clin. Infect. Dis..

[97] Bémer P., Léger J., Tandé D., Plouzeau C., Valentin A.S., Jolivet-Gougeon A., Lemarié C., Kempf M., Héry-Arnaud G., Bret L. (2016). How Many Samples and How Many Culture Media To Diagnose a Prosthetic Joint Infection: A Clinical and Microbiological Prospective Multicenter Study. J. Clin. Microbiol..

[98] Hischebeth G.T.R., Randau T.M., Molitor E., Wimmer M.D., Hoerauf A., Bekeredjian-Ding I., Gravius S. (2016). Comparison of Bacterial Growth in Sonication Fluid Cultures with Periprosthetic Membranes and with Cultures of Biopsies for Diagnosing Periprosthetic Joint Infection. Diagn. Microbiol. Infect. Dis..

[99] Jakobsen T.H., Xu Y., Bay L., Schønheyder H.C., Jakobsen T., Bjarnsholt T., Thomsen T.R. (2021). Sampling Challenges in Diagnosis of Chronic Bacterial Infections. J. Med. Microbiol..

[100] Bori G., Muñoz-Mahamud E., Garcia S., Mallofre C., Gallart X., Bosch J., Garcia E., Riba J., Mensa J., Soriano A. (2011). Interface Membrane Is the Best Sample for Histological Study to Diagnose Prosthetic Joint Infection. Mod. Pathol..

[101] Goh G.S., Parvizi J. (2021). Think Twice before Prescribing Antibiotics for that Swollen Knee: The Influence of Antibiotics on the Diagnosis of Periprosthetic Joint Infection. Antibiotics.

[102] Peel T.N., Dylla B.L., Hughes J.G., Lynch D.T., Greenwood-Quaintance K.E., Cheng A.C., Mandrekar J.N., Patel R. (2016). Improved Diagnosis of Prosthetic Joint Infection by Culturing Periprosthetic Tissue Specimens in Blood Culture Bottles. mBio.

[103] Pérez-Prieto D., Portillo M.E., Puig-Verdié L., Alier A., Gamba C., Guirro P., Martínez-Díaz S., Horcajada J.P., Trampuz A., Monllau J.C. (2016). Preoperative Antibiotic Prophylaxis in Prosthetic Joint Infections: Not a Concern for Intraoperative Cultures. Diagn. Microbiol. Infect. Dis..

[104] Burnett R.S.J., Aggarwal A., Givens S.A., McClure J.T., Morgan P.M., Barrack R.L. (2010). Prophylactic Antibiotics Do Not Affect Cultures in the Treatment of an Infected TKA: A Prospective Trial. Clin. Orthop. Relat. Res..

[105] Wouthuyzen-Bakker M., Shohat N., Sebillotte M., Arvieux C., Parvizi J., Soriano A. (2019). Is Gram Staining Still Useful in Prosthetic Joint Infections?. J. Bone Jt. Infect..

[106] Mirra J.M., Amstutz H.C., Matos M., Gold R. (1976). The Pathology of the Joint Tissues and Its Clinical Relevance in Prosthesis Failure. Clin. Orthop. Relat. Res..

[107] Zhao X., Guo C., Zhao G.-S., Lin T., Shi Z.-L., Yan S.-G. (2013). Ten versus Five Polymorphonuclear Leukocytes as Threshold in Frozen Section Tests for Periprosthetic Infection: A Meta-Analysis. J. Arthroplast..

[108] Sigmund I.K., McNally M.A., Luger M., Böhler C., Windhager R., Sulzbacher I. (2021). Diagnostic Accuracy of Neutrophil Counts in Histopathological Tissue Analysis in Periprosthetic Joint Infection Using the ICM, IDSA, and EBJIS Criteria. Bone Jt. Res..

[109] Bori G., McNally M.A., Athanasou N. (2018). Histopathology in Periprosthetic Joint Infection: When Will the Morphomolecular Diagnosis Be a Reality?. Biomed. Res. Int..

[110] Fink B., Makowiak C., Fuerst M., Berger I., Schäfer P., Frommelt L. (2008). The Value of Synovial Biopsy, Joint Aspiration and C-Reactive Protein in the Diagnosis of Late Peri-Prosthetic Infection of Total Knee Replacements. J. Bone Jt. Surg. Br..

[111] Fink B., Hoyka M., Blersch B., Baum H., Sax F.H. (2023). Graphic Type Differentiation of Cell Count Data for Diagnosis of Early and Late Periprosthetic Joint Infection: A New Method. Technol. Health Care.

[112] Mühlhofer H., Renz N., Zahar A., Lüdemann M., Rudert M., Hube R., Frommelt L., Ascherl R., Perka C., von Eisenhart-Rothe R. (2021). Diagnosis of periprosthetic joint infection: Development of an evidence-based algorithm by the work group of implant-associated infection of the AE-(German Society for Arthroplasty). Orthopade.

